# Care in the era of digital health: experiences from Norwegian general practitioners

**DOI:** 10.1080/02813432.2024.2437417

**Published:** 2024-12-05

**Authors:** Damoun Nassehi, Ellen Ramvi

**Affiliations:** Department of Caring and Ethics, Faculty of Health Sciences, University of Stavanger, Stavanger, Norway

**Keywords:** Primary health care, general practice, digital health technologies, care ethics, telehealth, qualitative research

## Abstract

**Objective:**

This study explores the experiences of General Practitioners (GPs) in Norway, examining the role of care in their practice and the impact of digital health technologies on their caregiving approach.

**Design:**

A qualitative study employing semi-structured interviews. The data was analysed by systematic text condensation.

**Setting:**

Conducted in various general practice settings within an urban region in southwestern Norway.

**Subjects:**

Eleven GPs were interviewed, chosen to reflect a diverse mix of ages, genders, and professional experiences.

**Results:**

The findings reveal that care occupied a central and multifaceted role in GPs daily practice, and that the care aspect of their practice was experienced as a source of personal fulfilment. Technologies such as Secure Digital Messaging (SDM) and Electronic Health Records could enhance the efficiency of care delivery and facilitate better management of patient interactions, however these technologies also present challenges in maintaining the depth of personal engagement that is central to the care ethics that characterise their caring role. The GPs emphasized the necessity of integrating digital tools in a way that supports the relational and ethical foundations of their caregiving role.

**Conclusion:**

This study underscores the enduring importance of care in general practice, even as digital technologies become increasingly prevalent. GPs maintain their caregiving roles by navigating the complexities of digital tools, highlighting the need for a careful balance between leveraging digital advancements and preserving the core values of care. The findings suggest a need for ongoing evaluation of digital tools to align them with the ethical foundations of care in general practice.

## Introduction

In Norway’s General Practitioner (GP)-based healthcare system, the continuity in general practice is associated with reduced use of outpatient specialist services, acute hospital admissions, and decreased mortality [1,2]. However, the work practices of GPs have evolved; for example, fewer GPs today make house calls compared to earlier [3], and the ‘Coordination Reform’ of 2012 involved expectations of increased municipal control and integration of general practitioners, as well as expectations to strengthen the GPs’ vertical relationship with specialist health services [4]. Additionally, there has been an increased interest and use of digital health technology [5–7]. During the time of this research, digital health technologies in GP offices primarily included secure digital messaging (SDM), teleconsultations, and electronic health records (EHRs). However, artificial intelligence-driven technologies are now emerging on the horizon. This technological evolution is driven by the goals of improving patient engagement, treatment and outcomes, aligning with broader trends of digitalisation and technological advancements in healthcare [8–14]. As GPs increasingly incorporate digital tools into their practice, questions arise regarding their caring role.

Existing literature on digital health in general practice frequently focuses on aspects such as patient engagement, efficiency, and outcomes of technology use, often sidelining the relational and caregiving aspects of the GP role [9,15–17]. Studies predominantly explore how digital tools enhance or challenge communication, workflow efficiency, and clinical decision-making, with less attention given to how GPs themselves experience and define care within their evolving professional roles [18–20]. Given the current shortage of GPs, understanding the GP role and how it is experienced is essential to addressing the GP crisis [21,22]. Moreover, there is a political debate on using digital technology to counteract the shortage of doctors by mainly making healthcare more efficient.

Based on the technological evolution, political recommendations, and previous research, it is therefore vital to uncover GPs’ experience of their role as caregivers and how digital technology affects this caring role [23]. As Pols [24] argues, care is more than technical efficiency—it is a relational practice shaped by interactions among technologies, routines, and human connections. While digital tools offer new ways to deliver care, they also transform these relational dynamics, raising important questions about what constitutes good care.

In this context, we understand ‘care’ as a multidimensional concept that encompasses not just the clinical aspects of treatment but also the relational and ethical dimensions of practice. It involves empathy, mutual respect, and trust, where both the GP and the patient engage in a reciprocal relationship marked by interdependence and shared vulnerability [25].

Given that the existing research has predominantly centred on patient perspectives and utilization [26], this study aims to fill the existing information gap by posing the following questions:

1. How do GPs experience and define the role of care in their professional practice?
2. How do digital health technologies affect GPs’ ability to provide care?

## Material and methods

From February 2022 to April 2023, spanning 14 months, the first author interviewed 11 GPs from various doctors’ offices about their thoughts on professional life, digital health technology, and their care for patients.

Semi-structured interviews were utilized to gain a deeper insight into each GP’s individual experiences. The collected data was analysed through systematic text condensation, following Malterud’s method [27]. This approach is primarily rooted in phenomenology and can be employed for a descriptive analysis to develop knowledge about the participants’ experiences.

### Settings and participants

This study is a component of a larger research initiative called Caring Futures [28], which seeks to create an innovative care ethics framework for technology-mediated care practices. The research took place in a region in southwestern Norway, where the majority of the population resides in urban areas. Typically, patients live close to their GP’s office, and most GPs, including those in this study, work in offices with one or more colleagues.

GP offices are mandated to use EHRs that connect to the Norwegian Health Network (NHN). Several distinct EHRs are provided by different private companies, such that GPs at the time of this study could choose between five different EHR-providers. All prescriptions and communications with other clinics and healthcare partners are conducted digitally. Most GPs provide digital portals for prescription renewals or SDM with the GP, while some GPs also offer video consultations. In addition to in-clinic consultations, which serve as the primary form of GP-patient contact, all GPs offer telephone consultations.

The first author employed a three-step process to recruit 11 participants. In November 2021, a formal letter was sent to all GPs in the region. Following this, a recruitment notice was posted on the informal Facebook Group ‘Allmenlegeinitiativet,’ which is frequently visited by the majority of Norwegian GPs. After receiving a few responses, the remaining participants were directly contacted by phone. The first author intentionally selected participants from a list of GPs in the region, aiming for a balanced representation in terms of age, gender, and years of experience as a physician to ensure a diverse and comprehensive perspective on the study topics. Very few GPs declined to participate (Table 1).

**Table 1.** Demographic data of participants (*n* = 11).

Age *(years)*	
Median	44
Range	35-63
Gender *(n)*	
Female	5
Male	6
Profession *(n)*	
General Practitioner	9
Resident in general practice	2
Experience as a physician *(years)*	
Median	15
Range	6-37
Place of medical degree *(n)*	
Norway	8
Other country	3
Workplace *(n)*	
City or Town	8
Village	3

### Data collection

The interviews featured open-ended questions, enabling physicians to reflect on their caring role with questions like: ‘How do you understand the term ‘care’’, ‘when are you experiencing that you provide good care’, ‘do you experience responsibility as a GP to provide care to the patients’. After this invitation to share their experiences with the meaning of ‘care’ in their practice the next sequence of questions was connected to digital technology: ‘What kind of technologies do you use, and how are these technologies affecting your care’. The interviews came to an end with a few questions about changes and their self-understanding such as: ‘Do you feel that you can be the professional you want to be in your work here’, ‘do you feel that the use of digital technology in your practice has an impact on how you function as a professional’.

The conversations were audio-recorded and transcribed word for word by the first author. After completing nine interviews, no new significant information emerged, so the data collection phase ended after 11 interviews. Each interview ranged from 36 to 74 min in length, with a median duration of 55 min and an average of 55 min and 49 s. The interviews primarily took place at the participants’ offices (*N* = 8), usually after working hours (*N* = 10). Two participants were interviewed at their homes, and one participant was interviewed at another office while on her way home from work. For the purposes of this study, the names of the GPs have been anonymised, and pseudonyms have been used in their place.

The first six interviews were conducted in person. However, during the interview period, GPs were experiencing high workloads, making it challenging to schedule appointments. Given that the initial interviews flowed smoothly, the researchers decided to conduct one, and subsequently, the remaining interviews *via* videoconference. Scheduling appointments with the GPs became much more manageable afterwards.

### Data analysis

The analysis was performed through a four-step process. First, the transcripts were read thoroughly through by both authors to gain a comprehensive impression. Second, the first author manually identified and sorted meaningful units corresponding to the study’s aim, followed by systematic decontextualization, where the text was removed from its context and matched with related text elements. The sorted and decontextualized text was read and discussed with the second author. Third, the resulting code groups were condensed into specific content. Finally, recontextualization was conducted and discussed by both authors, in which the content of each code group was described and summarized, and an analytical text with categories and subcategories was composed (Tables 2 and 3) [27].

**Table 2.** Overview of categories and subcategories.

*Category*	*Subcategory*
Central Role of Care	Individualizing treatments based on patient needs
	Compassion, active listening, and understanding
	Providing care in chronic and end-of-life stages
	Engaging with patients on multiple levels
GPs’ Professional Fulfilment	Joy in seeing patient progress
	Building long-term relationships
	Satisfaction from being a guide and stable presence
Impact of Digital Tools	Use of SDM for patient contact
	Enhancing care accessibility and efficiency
	Digital consultations as supplementary tools
Challenges with Digital Tools	Delays in communication response times
	Managing patient misinformation
	Data overload from health devices

**Table 3.** Example of analysis process using malterud’s method.

*Step*	*Example*
1. Total Impression	The initial reading highlighted the GPs’ emphasis on care, digital tool integration, and the challenges associated with technology in their practice.
2. Identifying Meaning Units	‘A lot of care lies in talking with the patient and showing that you have time and are listening’. (Oliver)
3. Condensation and Coding	Meaning Unit: Care involves listening and showing time for patients. Condensed Code: Compassion and active listening.
4. Synthesizing to Analytical Text	Subcategory: Compassion and active listeningCategory: Central Role of CareAnalytical Text: GPs emphasized that active listening and providing time for patients are key elements of care, going beyond merely administering treatments.

## Results

### Care is a Central part of general practice

Through the interviews with GPs, it became increasingly clear that care occupied a central and multifaceted role in their daily practice. These healthcare professionals highlighted the importance of individualising treatments to align with each patient’s unique life situation and personal preferences. This approach went beyond the traditional scope of medical treatment, focusing instead on a more holistic view of patient care.

One thing is to have compassion. I try to understand them in their current situation. […] A lot of care lies in talking with the patient and showing that you have time and are listening. It’s not just about giving treatment or medication that constitutes good care. (Oliver)

One of the significant aspects of this approach was the care provided to patients with chronic illnesses or those in the end-of-life stage. The GPs noted that this part of their job, though often challenging, was particularly meaningful. It involved not only managing medical needs but also providing emotional support, understanding the patient’s personal journey, and helping them and their families navigate through these difficult times. This kind of care required a deep understanding of the human condition, empathy, and the ability to connect on a level that transcends the medical.

[W]hen she comes to me, we talk a lot about what makes her sad. It then emerges that it’s partly due to the loss of qualities she had before. She has been a very caring person in the family and taken a lot of responsibility, but she can’t manage it anymore. And so, it’s about trying to say that now it’s not all your responsibility and you need to get others to help and let go of some of the burdens that you can’t do much about, and then there can be some good conversations. (Julia)

When GPs could not find an effective therapy for their patient, they would offer a different approach. In providing this type of care, GPs often found themselves playing various roles - from medical experts to counsellors, advocates, and sometimes even friends. They were required to engage with patients on multiple levels, considering not just their physical health but also their mental, emotional, and sometimes even spiritual well-being. This approach necessitated a keen understanding of the broader factors that affect health, including social, environmental, and psychological aspects. When dealing with their patients the GPs often found that there was a fluidity between providing traditional treatment and caring for their patients.

[…] she has started to talk about her childhood. She hasn’t done that before. Then she tells about abuse from her father. Something that no one has talked about before. (Emma)

Through sustained relationships with their patients, GPs cultivated a trusting environment that encouraged patients to disclose past traumas. This nurturing approach not only deepened GPs’ understanding of their patients’ conditions but also improved the effectiveness of the therapy provided.

### General Practitioners feel personally and professionally rewarded when giving care

For many GPs, the care aspect of their work was not just a professional responsibility but also a source of personal fulfilment. They described finding great joy in seeing their patients’ progress, whether in terms of health improvements or coping better with their conditions. Being a stable, caring presence in the lives of their patients was something that many GPs found deeply rewarding.

I really enjoy it when I get people to do things they hadn’t thought of, which are good for their health. So, and I hear it sounds a bit manipulative, but what I’m trying to do is to be a guide and help people on their journey. (Nicholas)

The doctors talked about the satisfaction derived from building long-term relationships with their patients, understanding their life stories, and helping them through various life stages and health challenges. This aspect of their work often provided a sense of accomplishment that went beyond the clinical outcomes. It was about making a difference in people’s lives, providing comfort, and sometimes being the main support system for patients who might otherwise feel lost in the healthcare system. The GPs also did not see their consultations as singular meetings but as part of a longer journey.

For example, I have a patient who is now dying. She lives in a care center. I have followed her all the way. I’ve made many house calls. When they sent me a message on Monday, I went to see her the following day. I prioritised using my lunch break to make a house call. And what I have received in return is that the patient appreciates it and is happy to see me. (Lily)

The GPs’ profound interest in their patients’ lives, stories, and overall wellbeing was a central motivation in their practice. They valued the opportunity to accompany their patients and families throughout life’s journey, from birth to death, whenever possible. This long-term engagement was not only a professional commitment but also a personal choice, with many GPs noting that their fascination with human stories influenced their decision to become a GP.

I feel that when I get a glimpse of who the patient is behind all the medical aspects, who they really are. You gain a holistic insight into others’ lives and ways of being. Yes, when you get a glimpse behind the facade and connect them with other family members and understand why they are the way they are. You get a very privileged view of many different things. (Sarah)

This human-centric approach underpinned the essence of general practice and was what many of the GPs found most rewarding about their profession. It was not just about treating diseases but about treating people, with all their complexities, fears, and hopes.

When I feel that I have gotten to know the patient and they have gotten to know me and we have […] a connection that makes them perhaps progress a bit. That the relationship in a way contributes to them feeling better in some way. (Sarah)

### General Practitioners use digital tools to enhance patient care and provider efficiency

During the interviews, the GPs noted that digital technologies had emerged as pivotal tools that enhance the care they provide. Video consultations and digital communication channels, for instance, had changed the way GPs interacted with their patients. These technologies had made healthcare more accessible, allowing patients to receive care without the need for physical visits to the clinic. In their daily routines, many GPs utilised digital tools such as SDM for patient communication. SDM worked as a secure email system directly connected to the GP’s EHR, simplifying the exchange of both messages and documents. One of the GPs also described how patients who had difficulty talking about emotional issues used SDM:

[T]here are some who have become very fond of it. And who quite often send messages that can be about emotionally difficult things. (Ben)

This shift towards digital healthcare had been particularly beneficial in reaching patients who might have difficulties visiting clinics, such as those with mobility issues, chronic conditions, or those living in remote areas. It also provided a flexible option for those who had busy schedules or could not afford to take time off work.

I think that we save the patients a lot of time. They can sit at work and often wonder about various things. It doesn’t necessarily have to be big issues. A lot can be solved digitally. […] It is less resource-intensive and more efficient. (Emma)

During the pandemic, video consultations became a temporary solution for many GPs. However, as practices adapted, most found telephone consultations to be simpler and more convenient for both patients and providers. While a few GPs continue to use video on occasion, it has largely been replaced by phone calls, which offer an easier and more immediate way to connect.

The systems also enabled patients to book appointments and refill prescriptions electronically, saving both time and resources while giving patients greater control over their health care. Patients appreciated the convenience and accessibility these digital options offer, which in turn has led to better care. Several physicians mentioned that SDM made it easier for parents to get advice on treatment for their children. Some of the GPs had experienced that SDM was helpful especially for providing care for those with psychological ailments, who had trouble coming to the GP office.

There are some with whom I manage to keep in better contact. I think that is very valuable. Patients with severe mental disorders correspond with me even when they are admitted. (Brian)

Some GPs had access to ultrasound or electronic stethoscopes, allowing them to provide more engaging and participatory care by inviting patients to see and hear their own health conditions, rather than merely being informed about them. Furthermore, the ability of patients to search for health information online and bring this knowledge into their consultations was seen as a positive step towards patient empowerment. It encouraged patients to be more involved in their healthcare and provided a starting point for more informed and productive discussions during consultations.

### Using digital health technologies poses dilemmas for GPs’ caring role

The integration of digital technologies in healthcare was not without challenges. GPs noted that while SDM was invaluable for certain situations, it could also lead to delays in treatment if messages were not promptly addressed. This issue was particularly pertinent in cases of acute health issues where immediate attention was required.

There was one [patient], an adult person, who got hit by a ball in the eye. And thought on a Sunday evening that ‘I need to get checked out. So, he sends an e-message Sunday evening thinking that I would refer him Monday morning. And I don’t think that eye was ruined, but coincidentally, I didn’t see that message until Tuesday. (Brian)

Additionally, the vast amount of health information available online could sometimes be overwhelming for patients and lead to anxiety and misinformation. Patients often came to consultations armed with information sourced from the internet, which sometimes could be inaccurate or misleading. This trend necessitated GPs to spend additional time debunking myths and calming unnecessary health fears.

I had a patient the other day who, before coming to an appointment, asked me through an e-consultation, to ‘read up on this link here’. And it wasn’t correct. […] If you are an arrogant doctor who just says: ‘You are wrong and this isn’t correct’, then they lose trust in you and won’t come to you. You often have to spend some time talking about what they have seen and explain why this might not be right, and then come up with another suggestion. (Oliver)

Another challenge noted by the GPs was the influx of data from digital health devices like heart rate monitors and fitness trackers. While this data could be useful, it also led to an information overload, making it challenging for GPs to sift through and identify relevant health concerns. This increased data management added to the workload of GPs, requiring them to spend more time on data interpretation and patient reassurance.

I lean towards the idea that with technology comes many new opportunities […] for patients who have a smartwatch or whatever it might be as a device that makes you track it better. [But] it becomes an ‘overload’ of data. (Mark)

The GPs observed that their patients often placed undue reliance on the data from their consumer devices, overshadowing their own body’s cues. For instance, several doctors recounted cases where patients were convinced they were sleep-deprived based solely on readings from their fitness trackers, despite not feeling tired or experiencing symptoms of sleep deprivation.

## Discussion

Our results underscore the central role of care in GPs’ professional lives and how this contributes to personal fulfilment. Building long-term relationships, understanding patients’ life stories, and guiding them through various life crises highlight the uniqueness of general practice. At the same time, interviewees in this study demonstrated considerable experience with a range of digital tools integrated into their daily routines, with SDM as a prominent example. This routine engagement reflects an adaptation to the digital era without becoming estranged from the potential of technology.

Among the most striking observations is the GPs’ ability to independently select technological solutions that enhance their relationship with patients and improve communication. This choice affords a certain degree of flexibility and adaptability, which is particularly important in an era where new digital solutions are continually introduced. The transition from video consultations back to telephone consultations post-pandemic illustrates this flexibility, where simplicity and accessibility for both doctor and patient were prioritised. The results also show that the care the GPs talk about extends beyond mere clinical tasks. It embraces a relational responsibility, where the GP not only provides treatment but also helps patients interpret and integrate digital health information in ways that respect their autonomy and promote trust. The physicians were able to retain their caring role while integrating various new technologies into their practice, even leveraging these tools as mediators of care in some instances. This seamless incorporation reflects their ability to use technology as an enhancement to, the relational aspects of their profession. By remaining sensitive to patients’ needs, taking time to contextualise data, and encouraging patients to trust their own bodily cues, they tailor care to each patient’s unique context and experiences.

Our findings demonstrate that the GPs’ desire and ability to care for their patients is not impaired by new digital health tools.

### Strengths and weaknesses of the study

This study’s strength lies in the detailed exploration enabled by semi-structured interviews, which uncover the depth of GPs’ experiences and insights regarding their role (care) in the digital age. This approach not only sheds light on the operational impacts of digital tools but also reveals their effects on the relational and ethical facets of patient care, aligning closely with our aim to understand care’s place in GPs’ professional practice.

The diversity of GP participants contributes to a comprehensive view of the caring role and the integration of digital technologies, offering a broad spectrum of perspectives. The study’s local setting provides a unique insight into how regional healthcare practices interact with digital advancements, enhancing our understanding of care’s evolving nature in a specific Norwegian context. However, this also presents a limitation, as findings may have limited transferability to broader or more diverse primary care settings. The unique regional characteristics and healthcare infrastructure may not fully represent other national or international contexts.

The first author’s medical background and experience as a GP facilitated easier access to and communication with other GPs, and a deeper understanding of their perspectives during interviews. Much of what the GPs expressed resonated with the first author’s own thoughts and understanding of the work as a GP. However, this familiarity could introduce a bias of assumed understanding. To mitigate this, the first author has actively engaged in discussions with the co-author, who is a professor in health science and has a background as a nurse as well as extensive experience which qualitative research, to ensure a balanced interpretation of the questions and findings.

The data collection was performed both in-person and *via* videoconference, a choice made from practical necessity. Although the first author did not observe significant differences in the depth of information gathered, digital interviews may inherently influence the quality of responses. The digital format might limit the richness of non-verbal communication and the natural flow of conversation, potentially resulting in less detailed or nuanced data compared to face-to-face interactions. This methodological variability introduces a possible limitation regarding the consistency and depth of the data collected across different formats. Moreover, relying solely on GP narratives could inadvertently overlook crucial patient-centric insights on digital technology’s impact on care experiences.

### Findings in relations to other studies

The integration of digital technology into general practice, as evidenced by our findings, must navigate the delicate balance between enhancing healthcare delivery and preserving the inherent humaneness of the GP-patient relationship. In this digital transformation, the essence of care, as delineated by Arborelius and Bremberg [29], must not be obscured. Our results show that the GPs advocating for a healthcare approach where patients are seen and treated as individuals beyond their clinical symptoms, underscoring the necessity for GPs to engage in conversations that extend into the personal realms of the patient’s life, such as family and work. We believe that this humanistic approach is critical to ensuring that digital communication platforms do not dilute the personal connection and understanding that form the bedrock of general practice.

The continuous, emphatic nature of the physician-patient relationship between GPs and their patients is considered central to the effectiveness of GPs [30,31]. It is through this relationship that the GPs in our study are capable of fluently choosing between therapeutic options and caring principles and applying the most appropriate option at the time for that particular patient. The GPs’ knowledge about and relationship with their patients is the main reason for why GPs should have a strong role in developing and choosing future digital technologies for primary care.

Furthermore, Frederiksen et al. [32] introduce the concept of attachment in the doctor-patient dynamic, emphasizing the profound need for continuous interpersonal relationships, particularly when patients feel vulnerable. This attachment need not be compromised in a digitally enabled healthcare setting. Instead, digital tools should be leveraged to reinforce the sense of security and trust inherent in the GP-patient relationship, ensuring that patients continue to feel understood and cared for, even in a virtual context. The GPs in our study show that they are quick to adapt to new technologies. As soon as a technology, such as SDM, has been deemed secure and incorporated into the EHR system so that it is easy to use, GPs are willing to use it [33]. Since SDM also reduces friction and increases effectiveness for patients, they are also willing to use SDM more regularly [34]. This creates the ‘perfect storm,’ explaining why SDM is so commonly used in our study and is likely to become one of the more important digital means of patient communication going forward. We see the rise of ‘over the counter’ physician service at pharmacies, ‘drop-in’ primary care clinics, and the use of ‘primary health care teams’ consisting of several different healthcare professionals [35] as contrary to the principles of continuity and closeness in primary healthcare. The GPs’ relationship with their patients, built over time, has a distinct advantage over other passing physician-patient relationships, such as at hospitals, where ‘treatment’ is prioritized, and ‘care’ can be reduced to a bare minimum [36]. Also, by reducing the possibility of building relationships there would be an increased risk of not being able to attract future GPs to the field as this was the main reason why the GPs in our study chose their profession [37].

Rudebeck’s [38] advocacy for person-centered care aligns with our findings, suggesting that digital technologies, when thoughtfully implemented, have the potential to deepen the GP-patient dialogue. By integrating comprehensive patient narratives and contextual data into healthcare interactions, digital tools can facilitate a more informed and empathetic approach to care, resonating with Rudebeck’s principles of understanding the patient’s personal narrative as central to effective healthcare provision.

### Meaning of the study

This study highlights the importance of maintaining the human element in care amidst the digital transformation, because while digital tools can enhance efficiency and access, they must be leveraged in a way that complements, rather than replaces, the personal engagements that lie at the heart of care in general practice. The nuanced approach of Norwegian GPs, selecting technologies that complement their caregiving role, underscores the need for a careful balance between leveraging digital advancements and preserving the core values of empathy, personal interaction, and understanding in patient care. It advocates for a patient-centered approach in the integration of digital tools, ensuring that technology serves as an aid rather than a barrier to the fundamental GP-patient relationship.

## Conclusion

This study reveals that while digital technologies, such as SDM, are becoming a common tool in general practice, GPs are keen on preserving the essence of care in their interactions with patients. The challenge lies in using digital tools in a way that maintains the quality of the GP-patient relationship. Despite the convenience offered by digital technologies, the need for a personal touch, empathy, and clear communication remains paramount. Ultimately, the successful integration of digital tools in general practice depends on GPs’ ability to uphold their ethical responsibilities, ensuring that technology complements rather than compromises the care essential to their professional role.
